# Supplementary material for: Learning curve and surgical outcome of robotic assisted colorectal surgery with ERAS program
Source: Sci Rep. 2022 Nov 29;12:20566. doi: 10.1038/s41598-022-24665-w (PMC9709162; doi:10.1038/s41598-022-24665-w)
Supplement: Supplementary file 1 — Supplementary Information 1. [file 41598_2022_24665_MOESM1_ESM.zip › LSC Raw data-Ñ[▒K/142/H_ñw╝╨░O▒Kñσ.pdf]

正式摘要(電子簽章：000000228218853)

W83 / W83 ☐ 英文標題(注意:無英文姓名-請補病人基本檔!) ☐ 【讀取】憑證

臺中榮民總醫院

出院病歷摘要

轉入號： 轉出號：

[redacted]

(8)流水編號	
(9)入院日期	110/10/18 (2021) 直腸外科 W83 - 081
(10)轉科日期	
(11)出院日期	110/10/26 (2021) 住院天數計 8 日

診斷

- 入院 :s-colon intramucosal adenocarcinoma, 20 FAV
  s/p colonoscopic biopsy
- 出院 :s-colon intramucosal adenocarcinoma, 20 FAV
  s/p colonoscopic biopsy
  s/p Laparoscopic Anterior resection and anastomosis on 2021/10/20
  sT3N0M0.

主訴

- Unexplained weight loss about 10kg in recent 6 months and bloody stool for 2 months.

病史

- This is a 78-year-old male patient with past history of
  1. HTN, on medication control
  2. Gout, on medication of colchicine
  3. Gastric ulcer, on medication of Omeprazole
  4. Ischemic stroke in 2019 with left side dysmetria by finger to nose test and knee heel shin test
  5. CKD

  He denied family history of colorectal cancer. He used to smoke 1PPD for 20+ years, quit for 10 years.

  According to the patient, he had unexplained weight loss about 10kg in recent 6 months and bloody stool for 2 months. Thus, he went to Puli VGH for help. L scope with biopsy was performed there on 2021/10/05 which recealed (1)s colon tubulovillous adenoma with high grade dysplasia, 30 cm FAV. (2)s colon intramucosal adenocarcinoma, 20 cm FAV. CT disclosed S colon mass lesion around 2.7cm and Rt kidney non function. Due to personal issue, he was transferred to our hospital for operation.

  He denied bowel habit change, nausea or vomiting, recent fever, abdominal discomfort.

  With the impression of s-colon intramucosal adenocarcinoma, 20 FAV, he was admitted for laparoscopic anterior resection and lung CT.

體檢發現

-
  - General appearance :
    - easy looking , conscious clear , without cardiopulmonary distress
    - Vital signs自動帶入date 體溫(BT) 脈搏(P) 呼吸(R) 血壓(BP) 身高(BH) 體重(BW) BMI
      20211018 35.8℃ 85次/分 16次/分 192/99mmHg 145cm 55kg 26.2
    - Vital signs: B.P. 192/99 mmHg , P.R. 85 /min , R.R. 16 /min , B.T. 35.8 ℃
  - HEENT :
    - Eyes: no anemic conjunctiva, no icteric sclera, normal EOM
    - ENT: no otorrhea, no rhinorrhea, no erythematous mucosa, no injected tonsil, no deformity
  - Neck: supple, freely movable, trachea midline,no deformity, no engorged jugular vein , no palpable thyroid or lymph node enlargement
  - Chest:
    - Inspection: Grossly normal, symmetric and fully expansion, no accessory muscle used, no operation scar, no spider angiomata, no gynecomastia
    - Palpation: no palpable mass or nodule, no subcutaneous emphysema, no tenderness
    - Ausculation: clear breathing sound
  - Heart:
    - Palpation: no thrill, no heave
    - Percussion: normal heart size
    - Ausculation: regular heart beat, normal S1, S2, no gallop, no murmur
  - Extremities: No deformity, full ranged of motion without limitation, no clubbing finger or nail cyanosis, no pitting edema, no ulcer or pigmentation, no obvious skin lesion, no varicose vein, no bone, joint or muscle tenderness

  CRS CONDITION

  - Karnofsky performance status: (100) Normal, no complaints, no evidence of disease

-
  - PE of Abdomen:
  - __1. Inspection: flat
  - __2. Ausculation: normoactive
  - __3. Palapation: no tenderness
  - __4. Percussion: dullness
  - CEA: pending ng/ml
  - Imaging Studies:
  - Colonoscope report: (1)s colon tubulovillous adenoma with high grade dysplasia, 30 cm FAV. (2)s colon intramucosal adenocarcinoma, 20 cm FAV
  - Sono , CT of abdomen and chest Films:CT disclosed S colon mass lesion around 2.7cm and Rt kidney non function
  - Figures:
    1. colon & rectum

手術日期及方法

- 手術日期及方法20211020 Laparoscopic Anterior resection and anastomosis - malignant

住院治療經過

- After admission, pre-operation survey showed no contraindication for the operation. Thus, the operation(Laparoscopic Anterior resection and anastomosis) was performed smoothly on 2021/10/20. The postoperative course was rather stable, we gradually titrate her diet from water to current warm II diet. There was no obstructive nor leakage sign. The patient was discharged later under stable condition. OPD follow-up was arranged on 11/01. We would explain the formal pathological report during OPD follow-up.

合併症

一般檢查

- 累積報告 :

DATE	TIME	ALB	TP	BIL.T	BIL.D	ALKP	AST	ALT	LDH	AMY	LIPASE	NH3	Ferritin	UA
1101018	1226	3.7		0.3	0.2	87	54	22	208					
1101020	1947	3.0		0.72				11						
1101025	0902			0.39				9						

DATE	TIME	WBC	RBC	HGB	HCT	MCV	PLT	BLST	NEUT	BAND	SEG	LYM	MON	EOS	BAS
1101018	1226	4520	4.51	13.2	40.0	88.7	281		71.0			19.9	6.4	1.8	0.9
1101020	1854	7640		11.8	35.6		196		92.3			6.7	0.8	0.1	0.1
1101025	0933	5120		11.2			212		66.0			15.0	10.2	8.0	0.8

DATE	TIME	NA	K	CL	BUN	CREAT	eGFR	CA	Mg	P	CRP	ESR	PCT	D-Dimer	LACTATE
1101018	1226	143	5.3		14	1.45		9.2							
1101020	1947	138	4.6		9	1.31	56.25	7.9							
1101025	0902	139	3.5		14	1.19	62.84	8.0							

DATE	TIME	GLUCOSE	HbA1C	CHOL	TG	HDL	LDL	KETONE	CK	CKMB	TRO-T	PT-P	APTT-P	NT-pro BNP
1101018	1226	85										11.9	31.0	

- 放射線報告 :檢驗項目 **:CT, LUNG** 報告日期 **:1101020**

  主　訴：
  Result:N
  Finding:
  CT of chest and mediastinum with IV contrast enhancement:
  > No obvious nodular lesion in both lungs. No CT evidence of lung metastasis.
  > Some small LNs in the middle mediastinum.
  > Multiple right renal cysts.
  > No enlargement of bil. adrenal glands.
  > Some small hepatic cysts.
  > DJD change of T-L spine with spur formation.
  Impression:
  No CT evidence of lung metastasis.

  檢驗項目 **:CHEST PA VIEW** 報告日期 **:1101019**
  主　訴：
  Result:N
  Finding:
  > Normal heart size
  > Mild increasing interstitial infiltration over bilateral lung field
  > fracture over left ribs
  > DJD change with spur formation of T-L spine.
  * suspicious nodular lesion over right lower lung
  Impression:
  As above description.

特殊檢查

病理報告

其它

- 檢驗項目 **:Bacterial Culture(Ordinary)** 檢體**:Sputum** 報告日期 **:1101025**

- 附註 ： Inadequate specimen
  陰性結果： Normal mixed flora presented.
  陽性結果： ISOLATED ORGANISM: COLONY COUNT:( )/ml
  (1)
  (2)
  (3)
  菌落量定義： P:Pure H:Heavy M:Moderate S:Slight moderate F:Few E:Enrich
  SUSCEPTIBILITY TEST: S:Susceptible I:Intermediate R:Resistant D: Susceptible Dose Dependent (SDD) NS: Nonsusceptible

	(1)	(2)	(3)	(4)
Ampicillin				
Cefazolin				
Clindamycin				
Clarithromycin				
Gentamicin				
Amikacin				
Chloramphenicol				
Ampicillin/Sulbactam				
Penicillin				
Gentamicin(120ug)				
Trimethoprim+Sulfamethoxazole				
Vancomycin				
Oxacillin				
Cefpirome				
Ciprofloxacin				
Minocycline				
Carbapen (IPM)				
Cefoperazone/Sulbactam				
Cefotaxime				
Ceftazidime				
Cefepime				
Ceftriaxone				
Flomoxef				
Levofloxacin				
Teicoplanin				
Piperacillin/Tazobactam				
Cefmetazole				
Aztreonam				
Tigecycline				
Amoxicillin/Clavulanic acid				
Cefoxitin				
Metronidazole				
Carbapen (MPM)				
Erythromycin				
Moxifloxacin				
Linezolid				
Ertapenem				
Colistin				
Daptomycin				

注意事項：

檢驗項目 :**GRAM STAIN** 檢體:**Sputum** 報告日期 :**1101023**
1) PMN 25 /LPF
2) Squamous epithelial cell >25 /LPF
3) Moderate Gram postive bacilli
4) Few Gram negative cocci
5) Few Gram postive cocci in chain
6)
7)
8)
備註:
@ FEW: 1 - 5, MODERATE: 6 - 30, HEAVY: > 30

出院時情況

- 3.改門診治療

出院指示

藥名	劑量	劑量單位	用法	頻次	數量	數量單位	現況	天數	單位
Colchicine tab 0.5mg	1	TAB	PO	QD	7	TAB	TH	7	天
OCAS Harnalidge 0.4 mg	1	TAB	PO	QDAC	7	TAB	TH	7	天
Sevikar tab 5/20mg	1	TAB	PO	QD	7	TAB	TH	7	天
Bokey cap 100mg	1	CAP	PO	QD	7	CAP	TH	7	天
(I I)Takepron OD tab 30mg	1	TAB	PO	QDAC	7	TAB	TH	7	天
Broen-C tab	1	TAB	PO	TID	21	TAB	TH	7	天
Ultracet tab	1	TAB	PO	Q6HPRN	28	TAB	TH	7	天

出院計劃

- 門診日期
  - date on 2021/11/01

主治醫師 2222H 林俊余 / 住院醫師 9727A 林順鴻

正式摘要(電子簽章：000000228218853)

W83 / W83

doctorRank:B
